# It is a question of equity: time to talk about children who are HIV‐exposed and “HIV‐free”

**DOI:** 10.1002/jia2.25850

**Published:** 2021-11-18

**Authors:** Amy L. Slogrove

**Affiliations:** ^1^ Department of Paediatrics & Child Health Faculty of Medicine & Health Sciences Stellenbosch University Worcester South Africa

1

Globally, in 2020, in addition to 37.7 million people living with HIV, 1.8 million of them being children <15 years, there were another 15.4 million children born to women living with HIV who did not acquire perinatal HIV, and are now surviving “HIV‐free” [1]. On the one hand, this number of children HIV‐exposed and HIV‐free is a cause for celebration. It reflects how access to interventions to treat maternal HIV and prevent vertical HIV acquisition have evolved magnificently over the last decade. On the other hand, this number is a cause for pause. It represents the persistently high HIV prevalence among adolescent girls and women in sub‐Saharan Africa [1]. *In southern African countries, an astounding 25% of all children are HIV‐exposed and HIV‐free* [2]. Yet, there has been little public discussion about the package of potentially adverse exposures experienced by children born to women living with HIV. These include unique exposures to HIV and antiretroviral drugs *in utero* and sometimes postnatally, and universal exposures that can adversely affect child outcomes irrespective of HIV exposure but that *occur more often* in children born to women living with HIV (Figure 1). This package of exposures is resulting in health inequalities among children born to women living with HIV even without HIV acquisition.

**Figure 1:**
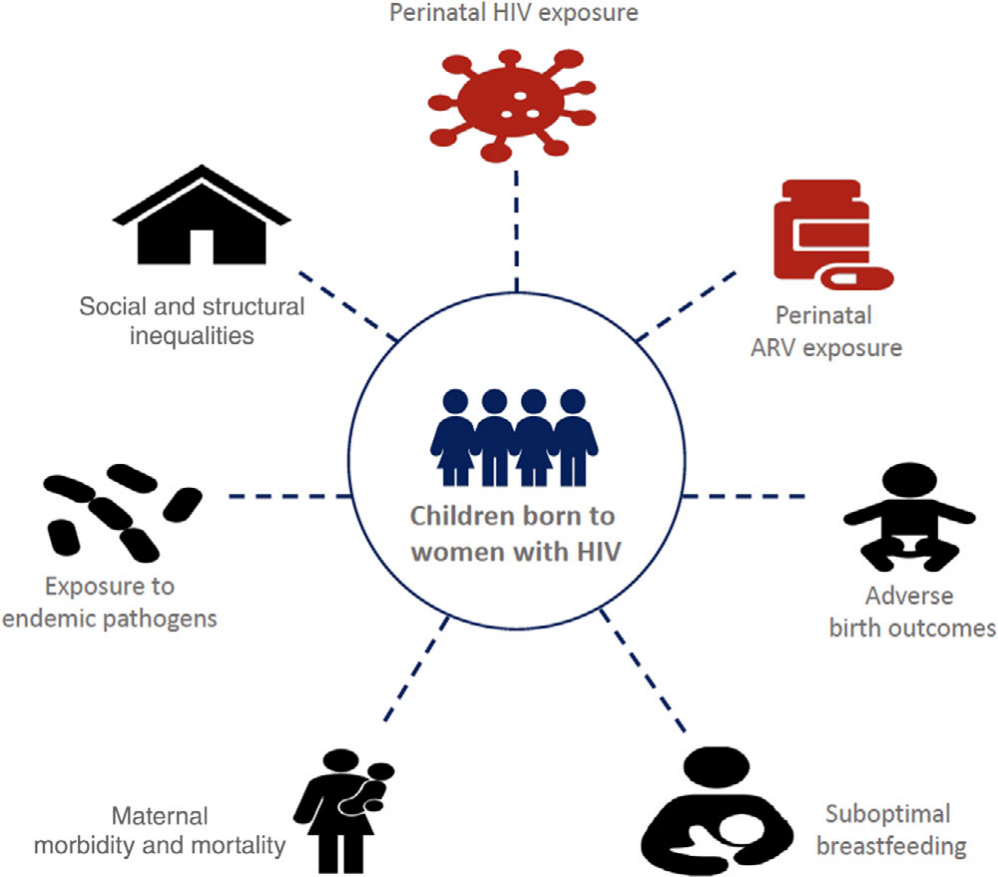
The package of risks for suboptimal early childhood health and development in children who are HIV‐exposed and HIV‐free. Note: ARV, antiretroviral; red‐coloured icons indicate risk factors unique to children HIV‐exposed and HIV‐free; black‐coloured icons indicate universal risk factors; social and structural inequalities refer to stigma, social exclusion, poverty and other adversities that can more often be experienced by families affected by HIV and impact on the health and developmental outcomes of children born to women with HIV.

Prior to antiretroviral therapy (ART) availability, it was accepted that adverse consequences to the health of pregnant women with untreated HIV and their unborn children were inevitable. An *in utero* environment altered by a severe viral infection cannot provide the conditions required for a foetus to optimally grow and develop. This was manifested early in the HIV pandemic by increased rates of stillbirth, foetal growth restriction and preterm birth [3]. As these children grew up, it became apparent that even when living without HIV they were more often sickly and not surviving as expected compared to children born to women without HIV [4, 5]. It was thought that factors like poor maternal health, inability to safely breastfeed and poverty were the most likely mediators of these outcomes [6, 7]. However, little was done to study whether they could possibly also be experiencing even worse health and developmental issues because of their HIV and antiretroviral exposures.

As ART became globally accessible, it was then assumed by providers and policymakers of national and global HIV programs that with improved maternal health and wellbeing, and the opportunity for safer breastfeeding in women on ART, pregnancy and child health outcomes would improve. However, recent evidence indicates that women living with HIV on ART are still at twice the risk of adverse birth outcomes, including stillbirth, preterm birth and foetal growth restriction compared to women without HIV [8]. It is as yet unclear whether ART or individual antiretrovirals are directly contributing to these adverse outcomes. However, we do know that maternal ART has not resolved the issue and that being born with one of these adverse birth outcomes *and* to a woman living with HIV multiplicatively impacts further on child outcomes even in the absence of vertical HIV acquisition. Compared to children who are HIV‐unexposed born preterm, those who are HIV‐exposed and HIV‐free born preterm experience higher rates of neonatal mortality, require neonatal intensive care twice as often and experience worse infant cognitive and motor developmental outcomes [9, 10, 11]. Similarly, infants who are HIV‐exposed with foetal growth restriction do not catch up their growth deficits during the first year of life as children who are HIV‐unexposed with foetal growth restriction do [12].

Recent data affirm that children HIV‐exposed and HIV‐free across the globe require hospitalization two to three times more often in early childhood, most frequently due to infectious diseases, such as viral and bacterial respiratory tract infections, invasive pneumococcal and group B Streptococcal infections as well as diarrhoeal disease [13, 14]. These children are also falling behind on expected early neurodevelopmental milestones, particularly in speech and language development [15, 16]. This can herald lifelong learning disabilities with poorer school completion as they age, which is linked to suboptimal income‐generating potential during adulthood as a result [17].

The limited available research has not yet fully elucidated the range and contributions of the individual components of the multifactorial package of exposures experienced by children born to women living with HIV. Determining whether HIV or antiretrovirals are playing a role is important in identifying potential interventions, but should not be necessary to determine whether or not we strive to resolve these inequalities in health, even if they are not biologically driven but driven instead by socially determined syndemics experienced by families affected by HIV.

With universal ART, the goal for adults living with HIV is one of equivalence in quality of life and life expectancy compared to adults living without HIV, and this goal should be no different for the children born to women living with HIV. We must look beyond only vertical HIV prevention and survival, which have been the primary focus of global maternal and child HIV programs for the last two decades. Global partners are coalescing around a new equity‐based goal of HIV‐free survival *plus* optimal early childhood development for all children born to women living with HIV [18]. Foundational to achieving this goal is to engage more directly in conversations with mothers and families in joint‐learning around how to optimize outcomes for all their children [2]. Mothers with HIV have voiced their distress at seeing but not understanding the relative developmental differences in their children compared to their peers, recognizing “that being HIV‐free is not always enough for our tender children” [19]. And they have expressed their bewilderment at being admonished by healthcare providers for even pointing out that there is something different about their children, including being scolded for their “ungratefulness” that their child is HIV‐free [19]. Simultaneously, mothers are concerned about preserving confidentiality for their families and the dilemma of “avoiding stigma‐by‐association for our children and securing the innocence of childhood unburdened by the complication of HIV” [20].

It is time for children who are HIV‐exposed and HIV‐free to be fully incorporated into the global HIV agenda. This means inclusion in goals and targets for the HIV pandemic that can be monitored through strengthened country‐level collection of child outcome indicators disaggregated by HIV exposure and not only HIV infection status. Research investments have started, but more are needed to fund partnerships in high HIV prevalence settings designed specifically to understand the mechanisms and potential interventions to ameliorate these adverse outcomes. It is the responsibility now of clinicians, researchers and policy‐makers to transparently and accurately share with families affected by HIV what is known, what remains unknown and what is being done to better understand the benefits, risks and consequences of *in utero* and early life exposure to antiretroviral drugs, HIV and an HIV‐affected environment. Continuing the narrow focus within the global HIV strategy on vertical HIV prevention alone and not pursuing equity in outcomes across the life course will be a major injustice to the “HIV‐free” generation and their families.

## COMPETING INTERESTS

The author declares no competing interests.

## AUTHOR'S CONTRIBUTIONS

ALS conceived of and wrote the Viewpoint.

## AUTHOR'S INFORMATION

ALS is a South African paediatrician and epidemiologist leading a research program centred around pregnancy and early life exposures and their impact on infant, child and adolescent outcomes with a particular focus on HIV and antiretroviral drug exposure. ALS provides a weekly specialist child health service for a structurally disadvantaged community in the rural Western Cape that informs her research approaches, and she is a strong advocate for local clinical and epidemiologic research skills capacitation among early career researchers.

## FUNDING

This work was supported by a career development award from the Fogarty International Center of the National Institutes of Health to ALS [grant number 1K43TW010683].
